# *Cajuputs candy *impairs *Candida albicans *and *Streptococcus mutans* mixed biofilm formation *in vitro*

**DOI:** 10.12688/f1000research.20700.2

**Published:** 2020-05-27

**Authors:** Siska Septiana, Boy Muchlis Bachtiar, Nancy Dewi Yuliana, Christofora Hanny Wijaya

**Affiliations:** 1 Department of Food Science and Technology, IPB University, Bogor, 16680, Indonesia; 2 Oral Biology and Oral Science Research Center, Faculty of Dentistry, Universitas Indonesia, Jakarta, 10430, Indonesia

**Keywords:** Cajuputs candy, essential oil, caries, mixed biofilm, Candida albicans, Streptococcus mutans

## Abstract

**Background:** *Cajuputs candy* (CC), an Indonesian functional food, utilizes the bioactivity of *Melaleuca cajuputi* essential oil (MCEO) to maintain oral cavity health. Synergistic interaction between *Candida albicans* and *Streptococcus mutans* is a crucial step in the pathogenesis of early childhood caries. Our recent study revealed several alternative MCEOs as the main flavors in CC. The capacity of CC to interfere with the fungus-bacterium relationship remains unknown. This study aimed to evaluate CC efficacy to impair biofilm formation by these dual cariogenic microbes.

**Methods: **The inhibition capacity of CC against mixed-biofilm comprising *C. albicans* and *S. mutans* was assessed by quantitative (crystal violet assay, tetrazolium salt [MTT] assay, colony forming unit/mL counting, biofilm-related gene expression) and qualitative analysis (light microscopy and scanning electron microscopy).

**Result: **Both biofilm-biomass and viable cells were significantly reduced in the presence of CC. Scanning electron microscopy imaging confirmed this inhibition capacity, demonstrating morphology alteration of *C. albicans*, along with reduced microcolonies of *S. mutans* in the biofilm mass. This finding was related to the transcription level of selected biofilm-associated genes, expressed either by *C. albicans* or *S. mutans*. Based on qPCR results, CC could interfere with the transition of *C. albicans *yeast form to the hyphal form, while it suppressed insoluble glucan production by *S. mutans*. G2 derived from Mojokerto MCEO showed the greatest inhibition activity on the relationship between these cross-kingdom oral microorganisms (p < 0.05).

**Conclusion: **In general, all CC formulas showed biofilm inhibition capacity. Candy derived from Mojokerto MCEO showed the greatest capacity to maintain the yeast form of *C. albicans* and to inhibit extracellular polysaccharide production by *S. mutans*. Therefore, the development of dual-species biofilms can be impaired effectively by the CC tested.

## Abbreviations

CC, *Cajuputs candy*; MCEO, *Melaleuca cajuputi* essential oil; G0, untreated biofilm, control group, without the addition of CC formula; G1, biofilm group treated with *Cajuputs candy* made with *Melaleuca cajuputi* essential oil from Pulau Buru; G2, biofilm group treated with *Cajuputs candy* made with *Melaleuca cajuputi* essential oil from Mojokerto; G3, biofilm group treated with *Cajuputs candy* made with *Melaleuca cajuputi* essential oil from Ponorogo; G4, biofilm group treated with *Cajuputs candy* made with *Melaleuca cajuputi* essential oil from Pasuruan; G5, biofilm group treated with *Cajuputs candy* made with *Melaleuca cajuputi* essential oil from Kuningan.

## Introduction

*Candida albicans* is the most prevalent fungus in oral microbiota ^1^. This opportunistic fungus grows as yeast, pseudohyphae, and hyphae based on environmental conditions ^2^. The hyphal form is relevant for its virulence as it allows penetration and invasion of epithelial cells ^3^.

*Streptococcus mutans* is a strong acidogenic and aciduric bacteria, defined as the major cause of dental caries. The critical virulence factor of *S. mutans* is its capacity to convert dietary sugars to produce an extracellular polysaccharide (EPS) matrix, mainly through glucosyltransferase enzymes (Gtfs) ^4^. EPS is the main building block of the biofilm. It can provide a binding site for colonization by other microbes and creates an acidic environment ^5, 6^.

Several studies have reported that *C. albicans* is frequently found with *S. mutans* in early childhood caries (ECC) ^7– 9^. The presence of both microbes indicates cross-kingdom feeding ^10^. Furthermore, GtfB from *S. mutans* plays a significant role in mediating this dual-species interaction ^11^. Their co-species interaction enhanced cell accumulation, biofilm formation, and Gtf gene expression ^8, 12^. Therefore, targeting the synergism of *C. albicans* and *S. mutans* in mixed biofilms has become a promising strategy for oral antimicrobial exploration ^13– 15^.

In accordance with the efficacy of essential oils as natural antimicrobial substances, *Cajuputs candy* (CC) has been developed using *Melaleuca cajuputi* essential oil (MCEO) as the main flavor ingredient. Previous work in our lab revealed the efficacy of CC in inhibiting biofilm formation by single oral microbes such as *S. mutans* (unpublished report) and *C. albicans* ^16^. This functional candy may have interfered with their synergistic relationship in dual-species biofilm ^5, 7, 8, 10^. Therefore, this study aimed to evaluate the capacity of CC to impair their symbiotic interaction. This finding will provide novel evidence for CC in interfering with the traits of cariogenic oral microorganisms.

## Methods

### Microbial strains and MCEO samples

A *C. albicans* and *S. mutans* Xc were used for this study. *C. albicans* was obtained from the Oral Biology Laboratory stock culture previously isolated from the patients with their consent in the dental hospital of Universitas Indonesia ^17^. *S. mutans* Xc was kindly provided by Prof. Yoshihisa Yamashita, Department of Preventive Dentistry, Kyushu University, Japan ^17^. They were maintained as glycerol stocks at -80°C in our laboratory. *C. albicans* was grown in Sabouraud dextrose broth (SDB) (Oxoid, UK) for 24 hours at 37°C. *S. mutans* was cultured in brain heart infusion (BHI) (Himedia Laboratories, India) for 24 hours under anaerobic conditions (10% CO _2_, 10% H _2_, 10% N _2_). The cell densities of each culture were quantified using total plate count on an agar medium.

Five essential oils were obtained. MCEO from Mojokerto, Ponorogo, Pasuruan, and Kuningan were provided by Perhutani Indonesia, whereas MCEO from Pulau Buru was obtained from local villages where they produce the MCEO on a small scale by home distilling. For this, approximately 300kg of sun-dried leaves of *Melaleuca cajuputi* are placed in the boiler of the distilling apparatus and hydrodistillation is performed for six hours. After passing through the condenser, the extracted oil is collected and separated from the residual water. A similar method was also used for the other extracts. The essential oil is stored in a dark bottle.

### CC preparation

The candies were prepared by mixing 98 g isomalt (Beneo-Palatinit GmbH, Germany), 0.1 g Acesulfame K (Anhui Jinhe Industries, China) and 0.1 g water ^18^. The mixed ingredients were heated to 150°C with continuous stirring. As the temperature decreased to 135°C, 820 µL MCEO and 180 µL peppermint oil (Brataco Chemika, Indonesia) was added and the dough was molded. Peppermint oil was used as a secondary flavor in addition to MCEO. To identify the most active MCEO, the MCEOs were varied among the candies. Pulau Buru was used as the targeted reference as it has been utilized from the beginning of our research series ^16, 18^ and needed to be replaced with other potent MCEOs due to its currently limited amounts. Four MCEOs were selected from our previous work as they had similar sensory characteristics to MCEO Pulau Buru ^19^. Five kinds of CCs were prepared using MCEO from different origins with Pulau Buru as the reference, and Mojokerto, Ponorogo, Pasuruan, and Kuningan as the alternative MCEOs.

### Mixed biofilm formation

A mixed biofilm was prepared on a 96-well plate by inoculating approximately 2 × 10 ^4^ colony forming units per milliliter (CFU/mL) of *C. albicans* suspended in SDB and 2 × 10 ^6^ CFU/mL of *S. mutans* in BHI in an equal suspension volume (50 µL). The well was previously coated with fetal bovine serum (FBS) (Biosera, South America) with one-hour incubation at 37°C. Similar with saliva, FBS coating aims to induce phenotype-associated *C. albicans* biofilm formation ^20– 22^. Supernatants were removed after a 90-minute incubation under anaerobic conditions ^15^. Then, 140µL of tryptic soy broth (Oxoid, UK) supplemented with 1% sucrose was added to each well followed by 60 µL of CC formula (each CC was dissolved in sterile distilled water (1:2 w/v) prior to the analysis). For the untreated control, the formula was replaced by 60 µL sterile phosphate-buffered saline (PBS). The biofilm group treated with CC made from Pulau Buru MCEO (as the reference) was represented as G1. Other treated groups G2, G3, G4, and G5 represented biofilms with the addition of CC made from Mojokerto, Ponorogo, Pasuruan, and Kuningan MCEOs, respectively. The untreated control (G0) was mixed biofilm without addition of the test CC formula. A light microscope equipped with a mobile camera (Primo Vert, Zeiss, Germany) was used to observe biofilm formation.

### Mixed biofilm analysis

The plates mentioned previously were incubated for zero, three, and 24 hours at 37°C under anaerobic conditions. The supernatants were aspirated and washed twice with 200 µL PBS. Attached biofilms were stained using 100 µL crystal violet (CV) 0.5% (v/v). Total biomass was extracted using absolute ethanol and absorbance at 600 nm was measured. This CV assay was performed in triplicate from two independent experiments.

Similar to the CV assay, the mixed biofilms on 96-well plates were washed twice with PBS after zero, three, and 24 hours of incubation at 37°C. Next, 50 µL of 5 mg/mL MTT (3-(4,5-Dimethylthiazol-2-yl)-2,5-diphenyltetrazolium bromide) were added for total cell viability analysis. The plates were incubated for three hours followed by tetrazolium salt extraction using 100 µL acidified isopropanol. After re-incubation for two hours at 37°C under anaerobic conditions, absorbance was measured at 600 nm. Three independent experiments were conducted in triplicate.

### Total plate count of *C. albicans* and *S. mutans*

The 24 hour biofilms on 96-well plates were washed twice with PBS. The biofilms at the bottom of the well were manually scraped and diluted with 300 µL PBS. The solutions obtained from each well underwent serial dilution and were grown for 24 hours at 37°C in separate media in triplicates. Sabouraud dextrose agar was used for *C. albicans*, whereas brain heart infusion agar was used for *S. mutans*.

### Morphology analysis of dual-species biofilm formation

A 24-well plate supplemented with 8 mm acrylic resin discs inside was used to grow the mixed biofilms. The biofilms were fixed by immersion in 1 mL of 2.5% glutaraldehyde for 1 hour followed by 20 minutes dehydration with each ethanol series (10, 25, 50, 75, and 90%). They were then immersed in 100% alcohol for one hour. The plates were dried at 37°C for 24 h ^20^. The mixed biofilm on the acrylic resin disc was analyzed using an FEI Quanta 650 Scanning Electron Microscope (SEM) (Thermo Scientific, Chicago).

### Mixed biofilm-related gene expression

The biofilm was harvested after 24 hours incubation. RNA was extracted using Trizol reagent (Sangon Biotech, China). cDNA synthesis was performed using ReverTra Ace qPCR RT Master Mix (Cat. No. FSQ-301; Toyobo, Japan) following the manufacturer’s protocol. cDNA concentration was measured using a Qubit RNA HS Assay Kit (Cat. No. Q32852; Thermo Fisher Scientific, USA). The PCR mixture contained 10 µL SensiFAST SYBR Hi-ROX (Cat. No. BIO-92020; Bioline Reagents, UK), 0.8 µL of the forward and reverse primer, nuclease free water, and 50 ng/mL of template-diluted cDNA to achieve a 20 µL final volume. Table 1 shows the list of primers used for *C. albicans* and *S. mutans* specific genes based on the literature ^13^. The PCR program for *C. albicans* genes was started with five minutes initial denaturation at 95 ^o^C, followed by 40 cycles of 15 seconds at 95°C and 60°C for one minute. For *S. mutans,* the PCR was run at 95°C for two minutes followed by 40 cycles of 95°C for five seconds and 60–61°C for 30 seconds. qRT-PCR was run on a StepOnePlus Real-Time PCR System (Applied Biosystems, USA). The relative gene expression was calculated as 2 ^-ΔΔCt^ and normalized to 18S rRNA and 16S rRNA for *C. albicans* and *S. mutans* genes, respectively.

**Table 1.** Primers used in qRT-PCR analysis of dual-species biofilm.

Primers	Sequences *
*ALS3*	F: CAACTTGGGTTATTGAAACAAAAACA R: AGAAACAGAAACCCAAGAACAACC
*HWP1*	F: GCTCCTGCTCCTGAAATGAC R: CTGGAGCAATTGGTGAGGTT
*YWP1*	F: GCTACTGCTACTGGTGCTA R: AACGGTGGTTTCTTGAC
*gtfB*	F: AGCAATGCAGCCAATCTACAAAT R: ACGAACTTTGCCGTTATTGTCA
*gtfD*	F: ACAGCAGACAGCAGCCAAGA R: ACTGGGTTTGCTGCGTTTG
16S rRNA	F: CCTACGGGAGGCAGCAGTAG R: CAACAGAGCTTTACGATCCGAAA
18S rRNA	F: CACGACGGAGTTTCACAAGA R: CGATGGAAGTTTGAGGCAAT

*Primer sequences were produced based on a previous study ^13^.

### Statistical analysis

Data analysis was performed using IBM SPSS Statistics 22 (IBM Corp., New York, USA). A one-way analysis of variance (ANOVA) followed by Duncan’s test (p <0.05) were used to analyze total biomass, cell viability, and CFU/mL. The means of gene expression were evaluated by Student’s *t*-test. All the graphs were produced using GraphPad Prism 8.0.2 (GraphPad Software, San Diego, California)

## Results

### Effect of CC on dual-species biofilm development

**Figure 1.**
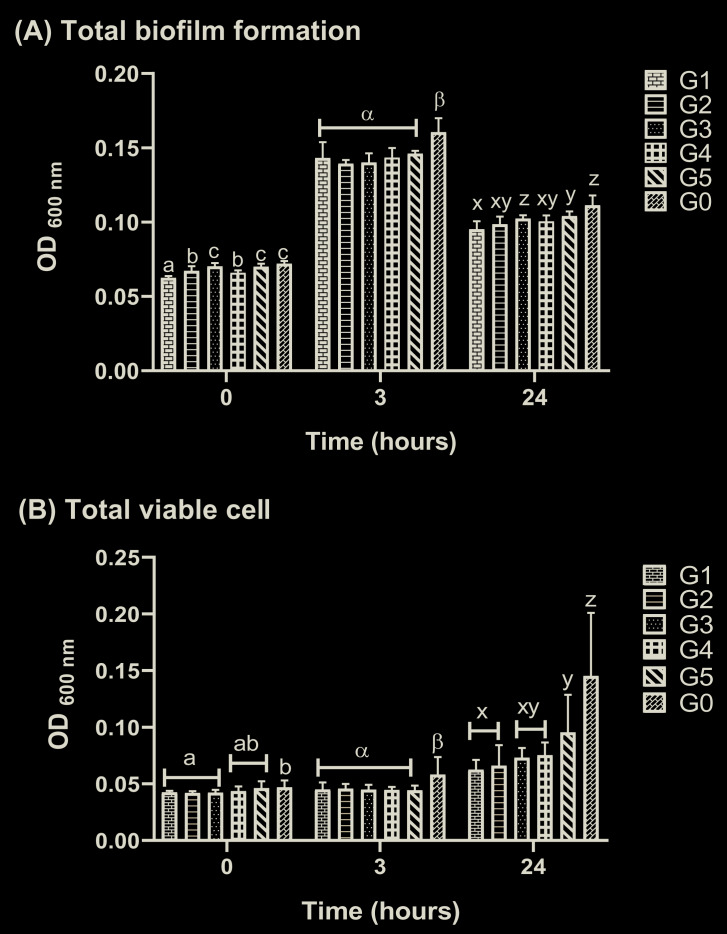
*Cajuputs candy* exposure inhibited *C. albicans* and *S. mutans* biofilm development: ( **A**) total biomass on *C. albicans* and *S. mutans* dual-species biofilm, evaluated by crystal violet (CV) assay; ( **B**) total viable cells on *C. albicans* and *S. mutans* dual-species biofilm based on MTT assay. The values were presented as mean and standard deviation of absorbance at 600nm. The letters on histogram represented the significantly different values compared to each other formula within the groups in 0, 3, or 24 hours according to Duncan’s test (p <0.05). G0: untreated control, biofilm group treated with *Cajuputs candy* made with *Melaleuca cajuputi* essential oil from different origins denoted by G1: Pulau Buru, G2: Mojokerto, G3: Ponorogo, G4: Pasuruan, and G5: Kuningan.

**Figure 2.**
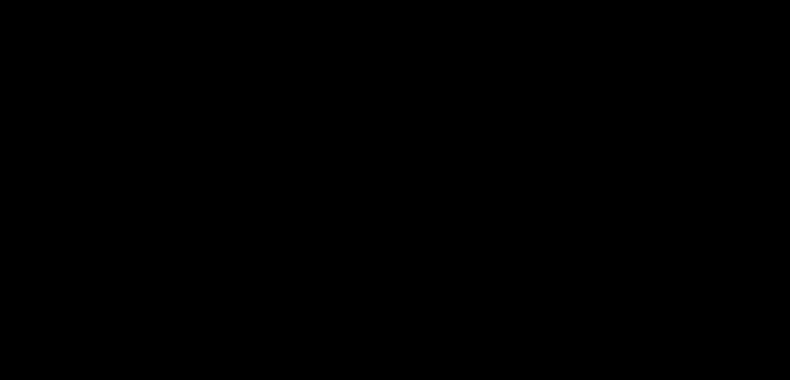
Total plate count (colony forming units/mL) of *C. albicans* and *S. mutans* on mixed biofilm ( *in vitro*): ( **A**) mean and standard deviation of the *C. albicans* colonies; ( **B**) mean and standard deviation of the *S. mutans* colonies. Different letters represented the significantly different values among the groups according to Duncan’s test (p <0.05). G0: untreated control, biofilm group treated with *Cajuputs candy* made with *Melaleuca cajuputi* essential oil from different origins denoted by G1: Pulau Buru, G2: Mojokerto, G3: Ponorogo, G4: Pasuruan, and G5: Kuningan.

All the CC formulae significantly inhibited biofilm development during the early-prematurity phase (0–3 hours) until the maturity phase (24 hours) ( Figure 1A). Total viable cell analysis showed comparable results ^23^. CC effectively suppressed both *C. albicans* and *S. mutans* viable cells (0–3 hours) ( Figure 1B) *.* Cell viability (24 hours) was also reduced in the presence of the CC formula, with G2 exhibiting the strongest capacity, similar to the reference group (G1). Figure 2 showed that CC exposure had similar efficacy against both microbes in single biofilm. G3, G4, and G5 did not interfere significantly with the number of *C. albicans* and *S. mutans* organisms, whereas G2 exhibited the highest inhibition capacity.

### Effect of CC on the morphology of dual-species biofilm

**Figure 3.**
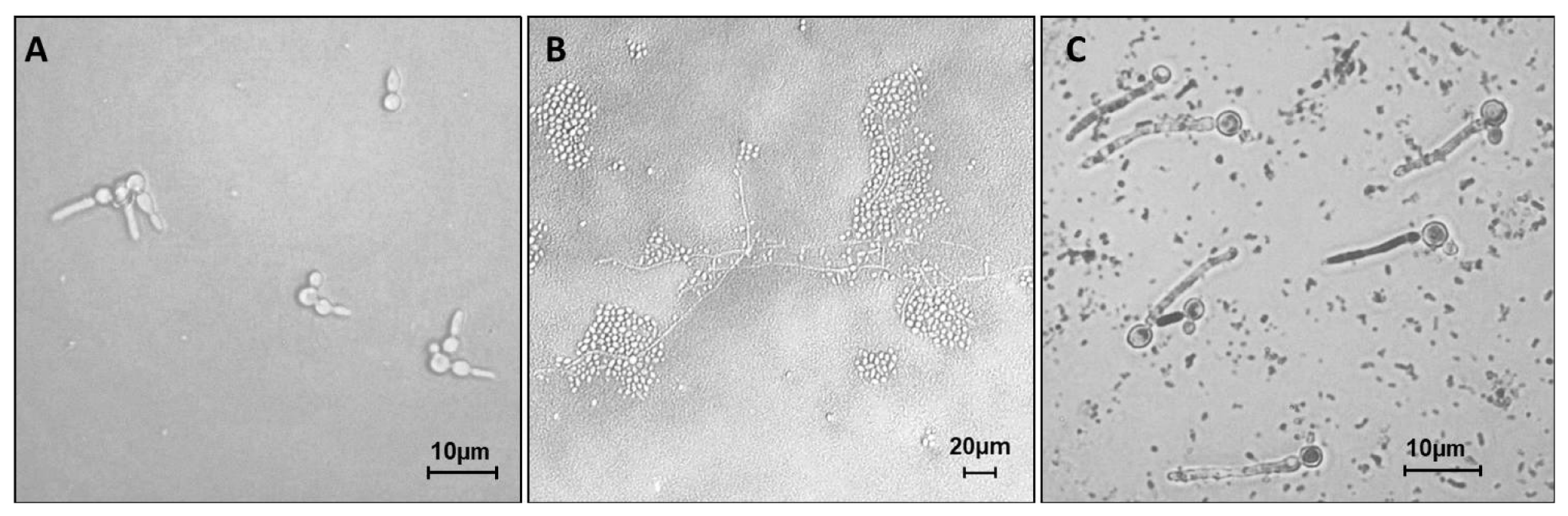
Light microscopy analysis of *C. albicans* and *S. mutans* interaction in mixed biofilm: ( **A**) germ tube formation in the first stage of biofilm development in 90 minutes (40× magnification); ( **B**) hyphal growth on untreated control of mixed biofilm after 24 hours incubation (20× magnification); ( **C**) dual-species interaction formed a *corn cob-like* structure for biofilm treated with *Cajuputs candy* (40× magnification). Grayscale color adjustment has been performed in order to clarify the figures.

Biofilm development started with the germ-tube formation of *C. albicans* in the 90 minutes before formula treatments ( Figure 3A). In the maturity stage (24 hours), the hyphal form of *C. albicans* dominated the biofilm, surrounded by *S. mutans* accumulation in the untreated control (G0) ( Figure 3B). A corncob-like structure ^24^ was observed in the mixed biofilm ( Figure 3C).

**Figure 4.**
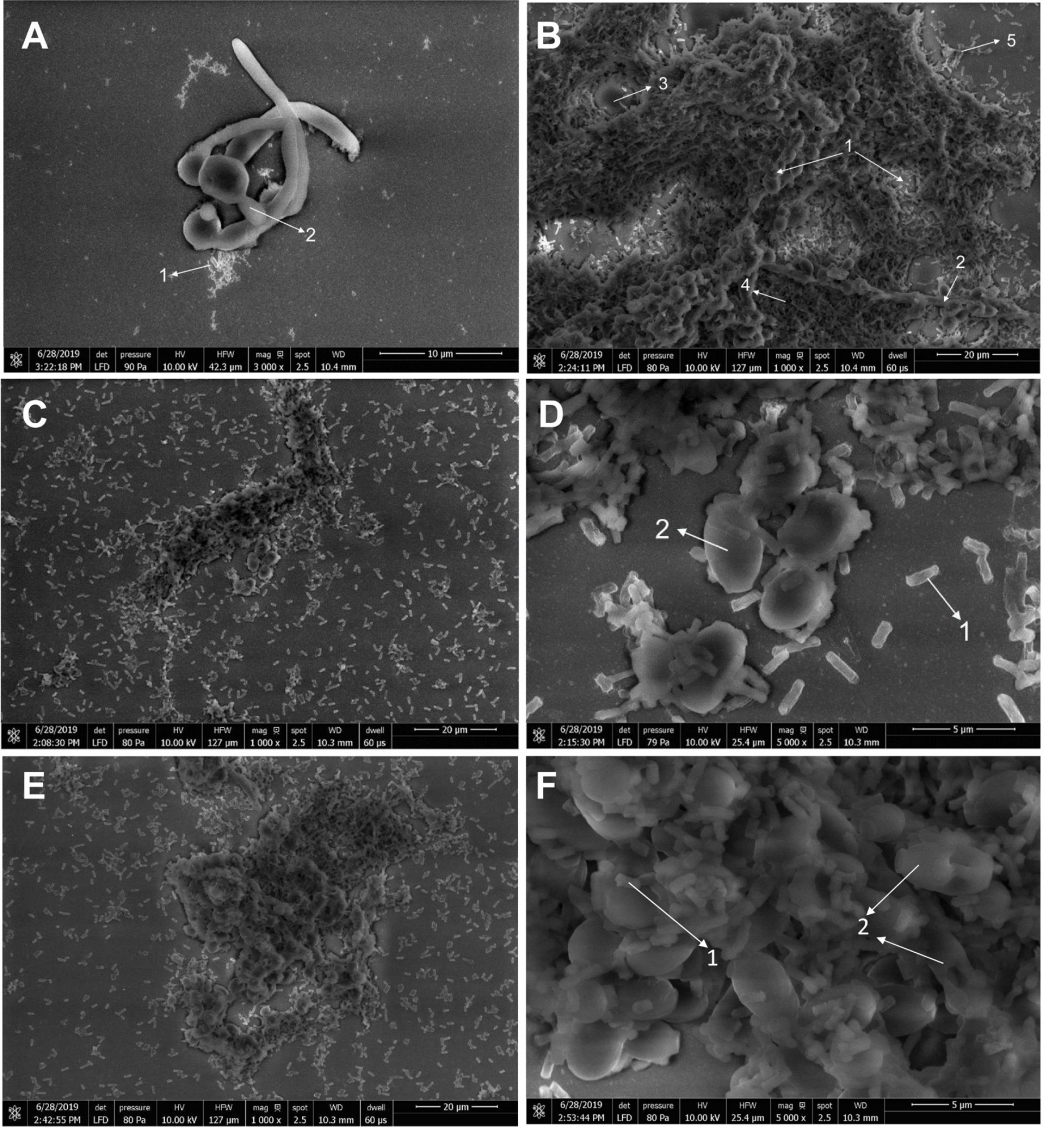
*In vitro* dual-species biofilm formation of *C. albicans* and *S.* mutans by scanning electron microscopy: ( **A**) initial germ-tube formation (3000× magnification); ( **B**) mixed biofilm of untreated control group (G0) (1000× magnification); ( **C**– **D**) mixed biofilm under G2 exposure (1000× and 5000× magnification, respectively); ( **E**– **F**) mixed biofilm under G5 exposure (1000× and 5000× magnification, respectively). The presence of *Cajuputs candy* reduced the hyphal cells of *C. albicans* and inhibited matrix production after 24 hours biofilm formation. (1. *S. mutans* cell; 2. *C. albicans* yeast and hyphal cells; 3. water channel; 4. extracellular polysaccharides matrix; 5. microcolony).

SEM analysis confirmed the germ tube formation in the first 90 minutes in which *S. mutans* was found close to *C. albicans* ( Figure 4A). As shown in Figure 4B, hyphal cells grew progressively in the untreated biofilm (G0), enclosed within the self-produced EPS matrix. This co-species population formed a complex structure within the biofilm. Interestingly, the presence of CC altered the architecture of the mixed biofilm. *C. albicans* tended to be maintained in yeast form, whereas *S. mutans* adherence to *C. albicans was* obviously reduced, especially for G2 ( Figure 4C–D). The microcolonies formed were not as many as those in the untreated control. However, exposure to G5 did not affect the interaction and a matrix-rich biofilm was still formed ( Figure 4E–F).

### Effect of CC on the expression of biofilm-related genes

**Figure 5.**
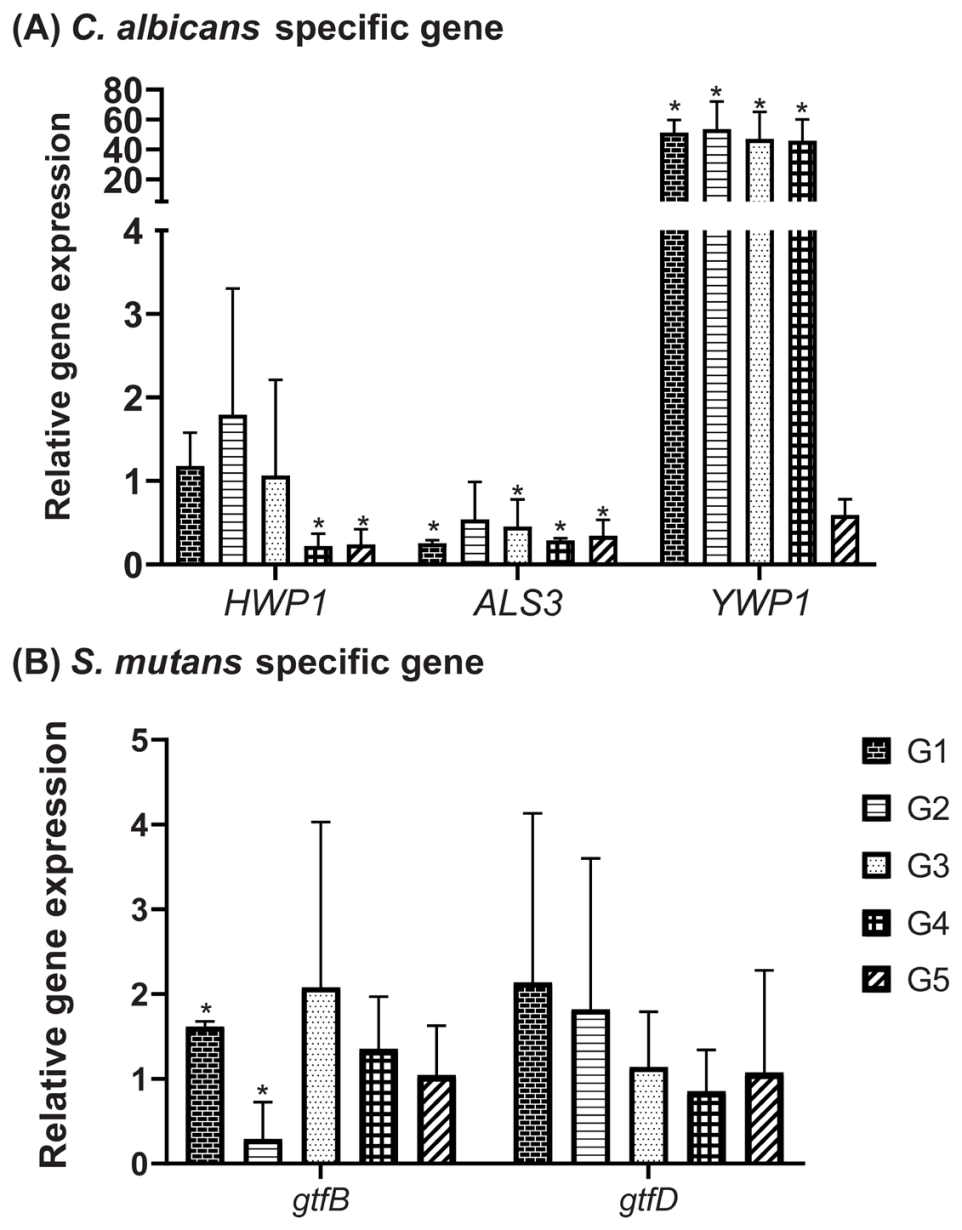
qRT-PCR assay of *C. albicans* and *S. mutans* biofilm-related genes: ( **A**) *C. albicans-*specific genes expression; ( **B**) *S. mutans* specific genes expression. An untreated control (G0) was defined as ‘1’. The values were shown as mean and SD. *Significantly regulated than the untreated control (G0) according to Student *t*-test (p<0.05). G0: untreated control, biofilm group treated with *Cajuputs candy* made with *Melaleuca cajuputi* essential oil from different origins denoted by G1: Pulau Buru, G2: Mojokerto, G3: Ponorogo, G4: Pasuruan, and G5: Kuningan.

All of the CC groups demonstrated significant downregulation of *ALS3,* the adhesion-specific gene of *C. albicans*. *HWP1*, which is responsible for hyphal filamentation, was still expressed in G1, G2, and G3. However, the expression of *YWP1*, the yeast-specific gene, was had a higher upregulation in almost all of the CC groups than other specific genes (HWP1 and ALS3) ( Figure 5A). This result confirmed the results of the SEM imaging, that CC exposure tends to maintain the commensal form of *C. albicans*.

As for *S. mutans* gene expression, the greatest downregulation was observed for *gtfB* in the mixed biofilm exposed to G2, whereas exposure to other formulas still allowed the expression of this insoluble glucan-specific enzyme ( Figure 5B). Regarding *gtfD* expression (the gene for the soluble glucan enzyme), none of the CC groups had a significant effect on gene regulation compared to the untreated control (G0).

## Discussion

CC is a lozenge that has been known as an emerging functional food in Indonesia. Further studies have shown its capability in maintaining oral cavity health due to the antimicrobial capacity of MCEO as its flavor against pathogenic oral microbes ^16, 18, 25^. In addition to the existing MCEO (PB), we successfully identified four additional MCEOs as potential CC flavors ^19^. However, the mechanism by which CC interferes with the relationship between the fungus and cariogenic bacteria ( *S. mutans*) remains unknown. CC consists of isomalt and peppermint oil in addition to MCEO as the main flavor. These ingredients were each added at the same concentration in all of the formulas. Hence, their effect can be assumed as background activity. So far, no studies have been performed to evaluate the efficacy of CC derived from several alternative MCEOs in attenuating the mixed biofilm of *S. mutans* and *C. albicans.* Our data show that all the CC groups showed a potent capacity in reducing the biofilm formation composed of these oral microflorae, as well as the viability of biofilm cells, until the biofilm reached its maturation stage. We observed that a higher total biofilm in the early prematurity phase (three hours) dominantly contributed to matrix production since cell viability was maintained at a low level. The colony number confirmed that viability reduction in the mature biofilm was contributed by the reduction in cell numbers of both microbes, with G2 demonstrating the strongest inhibition capacity, similar to our existing MCEO (G1) used as the reference ^18^.

The interkingdom interaction might begin in the first 90 minutes of biofilm growth, in which a corn-cob-like structure was observed (shown in Figure 3C). This result is in accordance with that of Zijnge *et al*. ^24^, who first found that *S. mutans* cells adhere to the hyphal cells of *C. albicans* to form this structure. This occurred due to the high affinity of *S. mutans* cells to the O-mannan group in the *C. albicans* cell wall ^7, 26^. Our study showed that G2 exposure intervenes in the *C. albicans* and *S. mutans* interaction, indicated by reduction in total biofilm and cell viability (CV and MTT assays, respectively). SEM imaging confirmed these quantitative results. The inhibition effect was related to the morphology alteration of *C. albicans* into the yeast form, inhibition of *S. mutans* adherence, and lack of microcolonies compared to the untreated control (G0).

The molecular mechanism underlying the CC inhibition capacity was explained by the expression patterns of selected biofilm-related genes. The adhesion trait of *C. albicans* was suppressed by *ALS3* downregulation when the CC formulas were present. As observed in this study, *HWP1* was still expressed in G1–G3. These two genes contribute to hyphal formation as the critical factors in *C. albicans* biofilm formation ^27^. However, the gene for the alteration from hyphal to yeast cell ( *YWP1)* was more dominantly expressed under CC exposure than the other specific gene (ALS3 and HWP1),, indicating that CCs tend to impair biofilm development by maintaining the yeast form of *C. albicans* with lack of adhesion and further filamentation. This was confirmed by observation of the hyphal form using SEM imaging ( Figure 4).

A parallel investigation of *S. mutans* genes showed that insoluble glucan production ( *gtfB*) was inhibited as an effect of G2 exposure, which showed greater inhibitory capacity compared to the G1 reference. In contrast, *gtfD* was still expressed, similar to the untreated control (G0) in all the CC groups. This means that these genes were still expressed in the biofilm. Furthermore, *gtfB* is one of the key factors for initiating dual-species interaction ^8, 11^. It has thus been found to bind *C. albicans* due to its low dissociation rate, resulting in strong and stable binding such as a covalent bond ^26^. Lower *gtfB* expression indicated a fewer matrix formation of *S. mutans* which important to form a polymicrobial biofilm with *C. albicans*, as shown by the CV and MTT assays in this study. This result also clearly explained the lack of a matrix on G2 SEM images ( Figure 4C and 4D).

Related to our finding, farnesol (quorum sensing molecule [QSM] of *C. albicans*) at low concentrations has reported inducing *S. mutans* growth besides GtfB ^10^. A lower concentration of farnesol could induce the hyphal form of *C. albicans*. QSMs are also produced by *S. mutans,* such as Autoinducer-2 (AI2), which is responsible for suppressing the inhibition capacity of farnesol. Another QSM of *S. mutans* is competence-stimulating peptide (CSP), which stimulates hyphal-to-yeast alteration ^28^. The result of this study showed that G2 caused a morphology alteration, which might also be correlated with the impairment of these QSMs. This inter-species signaling might induce the yeast form of *C. albicans*, which inhibits *S. mutans* cell accumulation. QSMs in the mixed biofilm was not measured quantitatively or qualitatively in our study. However, this assumption needs to be studied further.

MCEO, as a plant-based antimicrobial used in this experiment, significantly suppressed biofilm formation by reducing the cell number of both the microbes and also inhibited the total biomass production similar to other natural antimicrobials ^14, 15, 29^. Interestingly, the expression profile of morphology-related genes from *C. albicans* showed a comparable trend with the synthetic antimicrobial thiazolidinedione-8 (S-8) reported by Fieldman *et al*. ^13^. G2 also showed an additional activity of inhibiting *S. mutans* insoluble glucan production. This observation strengthens the potential of this formula to suppress mixed biofilm formation *in vitro*.

In general, all of the CC groups indicated potent inhibitory capacity against mixed biofilm formation. Mojokerto performed as the strongest MCEO in CC against the co-species *C. albicans* and *S. mutans* biofilm, comparable with the existing MCEO (Pulau Buru). This could be related to their similar metabolite composition as found in our recent work ^19^. MCEO from Mojokerto is dominated by 1,8-cineole (46.43%), caryophyllene (6.00%), α-terpineol (3.70%), γ-terpinene (3.09), and α-pinene (2.45%). The antibiofilm capacity of this MCEO could be related to these terpenic metabolites, as reported by several studies that essential oils from the *Melaleuca* genus have various antimicrobial activities ^30, 31^. Based on the previously published article, 1,8-cineole, a-terpineol, caryophyllene, linalool, terpinene-4-ol, and several other terpene compounds on MCEO were commonly reported as the responsible bioactive compounds on the MCEO antifungal and antibacterial activities ^32– 33^. Simşek and Duman ^34^ further reported the capacity of 1,8-cineole that increases the antimicrobial activity of chlorhexidine gluconate due to its synergistic effect and is expressed as a penetration enhancer. Moreover, Caryophyllene which most found in the MOJ also thought to be correlated with the effect of CC in the biofilm formation as it has been widely reported responsible for the antimicrobial activity ^35^. Nazzaro *et al*. ^36^ summarized their potential mechanisms such as cell wall degradation, affecting the quorum sensing system, and altering adherence capability.

## Conclusions

CC showed the ability to impair mixed *C. albicans* and *S. mutans* biofilm formation, with Mojokerto being identified as the most effective MCEO. Inhibition of the total biomass and cell viability were related with the candy’s capacity to maintain the commensal phenotype of *C. albicans* and to suppress insoluble glucan production by *S. mutans.*

## Data availability

### Underlying data

Open Science Framework: *Cajuputs candy* impairs *Candida albicans* and *Streptococcus mutans* mixed biofilm formation *in vitro*. https://doi.org/10.17605/OSF.IO/YT3HQ ^23^.

This project contains the following underlying data:

- Raw-unedited image files (original JPG files for images in Figure 3 and Figure 4)
- Raw Data of total biomass and cell viability.xlsx
- Raw Data of total plate count of each microbial strains on mixed biofilm.xlsx
- Raw Data of total qPCR assay on specific genes.xlsx

Data are available under the terms of the Creative Commons Zero "No rights reserved" data waiver (CC0 1.0 Public domain dedication).
