# Supplementary material for: Cajuputs candy impairs Candida albicans and Streptococcus mutans mixed biofilm formation in vitro
Source: F1000Res. 2020 May 27;8:1923. Originally published 2019 Nov 15. [Version 2] doi: 10.12688/f1000research.20700.2 (PMC7269370; doi:10.12688/f1000research.20700.2)
Supplement: Peer review history

# Peer review: Reviewer response for version 2

**DOI:** 10.5256/f1000research.26745.r63983

**Published:** 2020-06-02

**Authors:** Mohd Hafiz Arzmi, Hasna Ahmad

**Affiliations:** 1 Fundamental Dental and Medical Sciences Department, Kulliyyah of Dentistry, International Islamic University Malaysia (IIUM), Kuantan, Malaysia; 2 Kulliyyah of Allied Health Sciences, International Islamic University Malaysia (IIUM), Kuantan, Malaysia

I have no further comment to make.

Is the work clearly and accurately presented and does it cite the current literature?

Yes

If applicable, is the statistical analysis and its interpretation appropriate?

Partly

Are all the source data underlying the results available to ensure full reproducibility?

Yes

Is the study design appropriate and is the work technically sound?

Yes

Are the conclusions drawn adequately supported by the results?

Partly

Are sufficient details of methods and analysis provided to allow replication by others?

Yes

Reviewer Expertise:

Oral microbiology and immunology; natural products; polymicrobial biofilms

We confirm that we have read this submission and believe that we have an appropriate level of expertise to confirm that it is of an acceptable scientific standard.

# Peer review: Reviewer response for version 1

**DOI:** 10.5256/f1000research.22766.r58901

**Published:** 2020-02-17

**Authors:** Dikdik Kurnia

**Affiliations:** 1 Department of Chemistry, Faculty of Mathematics and Natural Sciences, Padjadjaran University (UNPAD), Bandung, Indonesia

1. The main structure of the research and main data are according and relevant, with all references, and showed the new findings effect on mix biofilm formations.
2. As an addition if possible, in table 1, the effect of CC against individual bacterial biofilm can be added as a reference or control.
3. As a suggestion, in the discussion, it would be better to explain the bioactive compounds of  *Melaleuca cajuputi *reported in other published papers. In this way, we can predict the active constituents of *M. cajuputi* that show an effect to biofilm formation.

Is the work clearly and accurately presented and does it cite the current literature?

Yes

If applicable, is the statistical analysis and its interpretation appropriate?

Yes

Are all the source data underlying the results available to ensure full reproducibility?

Yes

Is the study design appropriate and is the work technically sound?

Yes

Are the conclusions drawn adequately supported by the results?

Yes

Are sufficient details of methods and analysis provided to allow replication by others?

Yes

Reviewer Expertise:

Bioactive Natural Products

I confirm that I have read this submission and believe that I have an appropriate level of expertise to confirm that it is of an acceptable scientific standard.

# Response

**Published:** 2020-05-21

**Authors:** Christofora Hanny Wijaya

**Affiliations:** IPB University, Indonesia

Dear reviewer,

Please accept our sincere apology for this delayed response. We do appreciate the opportunity to revise our paper entitled, **“ *Cajuputs candy *impairs *Candida albicans *and *Streptococcus mutans* mixed biofilm formation *in vitro*** **”. **We also extend our deep gratitude to the reviewers for providing valuable comments and correction on our paper. We have read and understood the comments from the reviewers. Please find below our point-by-point response to the reviewer’s comments and concerns. We hope our additional explanations will be able to answer the reviewer's inquiry.

We strived to improve the quality of our paper based on the guidance and constructive suggestion provided by the reviewers. We hope that the revisions will be sufficient to make our paper worthy for publication in F1000 Research. We would like to thank you for your hearty support and willingness to find the parts of our paper needing corrections or improvement. Thank you again for your consideration, suggestions, and insightful feedback in order to greatly improve our paper.

Sincerely yours,

Authors: Siska Septiana, Christofora Hanny Wijaya*, Boy Muchlis Bachtiar and Nancy Dewi Yuliana.

**Reviewer Comments and Author Responses**

**Comment 1:** *The main structure of the research and main data are according and relevant, with all references, and showed the new findings effect on mix biofilm formations.*

**Response to reviewer’s comments 1**:

Thank you so much for your positive feedback. We greatly appreciate your reviews in our paper.

**Comment 2:** *As an addition if possible, in table 1, the effect of CC against individual bacterial biofilm can be added as a reference or control.*

**Response to reviewer’s comments 2**:

Thank you for the comments. Your suggestion is reasonable, unfortunately, we couldn’t conduct an additional experiment due to the limited sample. However, as reported in the previous study (unpublished report, Wijaya et al. 2014), CC had been proven to has antibiofilm activity on a single biofilm either on *S. mutans *or on *C. albicans*. Our study was then conducted as further evaluation of CC against synergistic interaction between the organisms. Therefore, we focused on the effect of CC against the dual-species biofilm, and the effectivity of CC against individual strain was further evaluated using *Total Plate Count*, as shown in Figure 2.

**Comment 3:** *As a suggestion, in the discussion, it would be better to explain the bioactive compounds of Melaleuca cajuputi reported in other published papers. In this way, we can predict the active constituents of M. cajuputi that show an effect to biofilm formation.*

**Response to reviewer’s comments 3**:

Thank you for your valuable suggestion, your suggestion would enrich our paper. We have added the additional information regarding the possible bioactive compound. The new version of the paragraph can be found as the sixth sentence in the last paragraph in the discussion section:

*Based on the previously published article, 1,8-cineole, a-terpineol, caryophyllene, linalool, terpinene-4-ol, and several other terpene compounds on MCEO were commonly reported as the responsible bioactive compounds on the MCEO antifungal and antibacterial activities *(Rini et al. 2012; Wińska et al. 2019) *. *Simsek and Duman (2017) *, further reported the capacity of 1,8-cineole that increases the antimicrobial activity of chlorhexidine gluconate due to its synergistic effect and is expressed as a penetration enhancer. Moreover, Caryophyllene which found most in the MOJ sample *(Septiana et al. 2020) *also thought to be correlated with the effect of CC in the biofilm formation as it has been widely reported responsible for the antimicrobial activity *(Yoo and Jwa 2018) *.*

References:

- Rini P, Ohtani Y, Ichiura H. 2012. Antioxidant, anti-hyaluronidase and antifungal activities of *Melaleuca leucadendron Linn*. leaf oils. *J. Wood Sci.* 58(5):429–436. doi:10.1007/s10086-012-1270-x.
- Septiana S, Yuliana ND, Bachtiar BM, Putri SP, Fukusaki E, Laviña WA, Wijaya CH. 2020. Metabolomics approach for determining potential metabolites correlated with sensory attributes of Melaleuca cajuputi essential oil, a promising flavor ingredient. *J. Biosci. Bioeng.* 129(5):581–587. doi:10.1016/j.jbiosc.2019.12.005.
- Simsek M, Duman R. 2017. Investigation of effect of 1,8-cineole on antimicrobial activity of chlorhexidine gluconate. *Pharmacognosy Res.* 9(3):234–237. doi:10.4103/0974-8490.210329.
- Wijaya CH, Rachmatillah AF, M. Bachtiar B. 2014. Inhibition of *Cajuputs Candy* Toward the Viability of *Candida albicans *by using In Vitro Assay. *J. Teknol. Dan Ind. Pangan* 25(2):158–167. doi:10.6066/jtip.2014.25.2.158.
- Wińska K, Mączka W, Łyczko J, Grabarczyk M, Czubaszek A, Szumny A. 2019. Essential Oils as Antimicrobial Agents—Myth or Real Alternative? *Molecules* 24(11):1–20. doi:10.3390/molecules24112130.
- Yoo HJ, Jwa SK. 2018. Inhibitory effects of β-caryophyllene on *Streptococcus mutans* biofilm. *Arch. Oral Biol.* 8842–46. doi:10.1016/j.archoralbio.2018.01.009.

# Peer review: Reviewer response for version 1

**DOI:** 10.5256/f1000research.22766.r58903

**Published:** 2020-02-05

**Authors:** Mohd Hafiz Arzmi, Hasna Ahmad

**Affiliations:** 1 Fundamental Dental and Medical Sciences Department, Kulliyyah of Dentistry, International Islamic University Malaysia (IIUM), Kuantan, Malaysia; 2 Kulliyyah of Allied Health Sciences, International Islamic University Malaysia (IIUM), Kuantan, Malaysia

The manuscript elucidates the activity of Cajuputs to *Candida albicans* and *Streptococcus mutans *in mixed biofilms. The effect was assessed using CV assay, MTT, CFU, genes expression and SEM. The authors claim that Cajuput inhibits biofilm and production of polysaccharide by *S. mutans.*

 This study is fascinating; however, major revision is suggested to improve the manuscripts. The following are the comments for authors to consider:

**Section: Introduction**

- It would be best if the authors can include the objective/s and the hypothesis of the study in the introduction section.

**Section: Methodology**

 Microbial strains and MCEO sample:

- Did C. albicans was grown in aerobic or anaerobic?  
- For MCEO Pulau Buru, what is the temperature in the boiler? If it is 100 degrees Celcius, won't it affect the active compound of the extract? Is this the same method used for the other extracts? If yes, it would be great if you mention that in the paragraph.

CC preparation:

- Won't 150 degrees Celcius affect your active compound? If that is the method, it would be better if you can put a citation on the method. As the expertise in natural product, it seems the temperature is too high for an extract.  Unless if the method is commonly used in the preparation by the local. 

Mixed biofilm formation:

- When you prepare the mixed biofilm, you did mention that you grew *C. albicans* in SDB and *S. mutans* in BHI in equal volume. Did you mix both media to prepare biofilm? If not, please make it clear in your text.  
- Why did you coat the well with fetal bovine serum? Better if you can explain why because that is not the standard method used in polymicrobial biofilms study.
- For you control G0, you mentioned the biofilm was developed without CC. What is the final volume of the well? Did that remain to 200 uL or 140 uL of TSB? If it was 140 uL, how do you compare with other wells which the total volume is 200 uL?

Morphology analysis of dual-species biofilm formation:

- Why did you choose acrylic as the surface to form a biofilm, and how do you relate this with well that have a different surface?

**Section: Results**

 Effect of CC on dual-species biofilm development:

- 'Susceptibility' is generally referred to as the antimicrobial activity of the extract. In your research, you are emphasizing on biofilm (Figure 2) and not the antimicrobial effect of the extract.  
- Figure 1. In the legend, the author mentioned that different letters represent significantly different. What does the author comparing too? Significant compared to what? Better to mention.
- What is a, b and c represent?  E.g. 'a' in Figure 1A doesn't show significant at 3 hours. The figure seems confusing.  I would suggest improving the figure to ensure understanding of readers. Similar to figure 2.

Effect of CC on the morphology of dual-species biofilm:

- Figure 3A doesn't look corncob to me. Please read this article:
  The microbial infection of biomaterials: A challenge for clinicians and researchers. A short review. Journal of Applied Biomaterials & Biomechanics 2005; Vol. 3 no. 1: 1-10 ^1^
- What do you mean by microcolonies with reference to the figure? Please explain.
- How do you determine significant from SEM image? What was the statistical analysis did you use to claim the significant of your SEM? Did you use image J or any software to measure the biofilm based on your SEM?  If yes, better to mention in your methodology. If not, please remove the 'significant'.

Effect of CC on the expression of biofilm-related genes:

- The author mentioned 'upregulated dominantly'. What do you mean by dominantly and compare to what? G0? G1?
- What do you mean by 'to maintain the commensal form of *C. albicans*'?

**Section: Discussion**

- The author claims that all CC groups reduce biofilm biomass. What is the author comparing too? Based on my observation, there is no significant difference in total biomass for 24 h and 0 h when all formulation are compared to G0. Please check your statistics.
- Figure 4D. This doesn't look cocci shape to me. More like bacilli/rod. I hope you did verify your sample prior to the experiment. Attached is the SEM of commonly seen *Streptococcus mutans* under SEM.
https://www.nature.com/articles/s41598-017-08558-x ^2^
- The author mentioned that lower *gtfB* indicate the fewer binding site. Glucosyltransferases (Gtfs) are enzymes and not a 'binding site'. This is too speculative. Please consider to rephrase or to remove the statement.
- The author also emphasised a lot on quorum sensing molecule which is not studied in the manuscript. The author also mentioned that QSM is not measured quantitatively. This statement can be misinterpreted none of the section showed the authors have conducted study on QSM either quantitatively or qualitatively. Please consider rephrasing.

Overall, this is a good study; however, more improvement is needed to fit with F1000research, a Q1 journal.

Is the work clearly and accurately presented and does it cite the current literature?

Yes

If applicable, is the statistical analysis and its interpretation appropriate?

Partly

Are all the source data underlying the results available to ensure full reproducibility?

Yes

Is the study design appropriate and is the work technically sound?

Yes

Are the conclusions drawn adequately supported by the results?

Partly

Are sufficient details of methods and analysis provided to allow replication by others?

Yes

Reviewer Expertise:

Oral microbiology and immunology; natural products; polymicrobial biofilms

We confirm that we have read this submission and believe that we have an appropriate level of expertise to confirm that it is of an acceptable scientific standard, however we have significant reservations, as outlined above.

## References

[1] : The microbial infection of biomaterials: A challenge for clinicians and researchers. A short review. *J Appl Biomater Biomech*.3(1) :1-10 20799234

[2] : Spontaneous detachment of Streptococcus mutans biofilm by synergistic effect between zwitterion and sugar alcohol. *Scientific Reports*.2017;7(1) : 10.1038/s41598-017-08558-x 10.1038/s41598-017-08558-x 28808327PMC5556044

# Response

**Published:** 2020-05-21

**Authors:** Christofora Hanny Wijaya

**Affiliations:** IPB University, Indonesia

Dear reviewers,

Please accept our sincere apology for this delayed response. We do appreciate the opportunity to revise our paper entitled, **“ *Cajuputs candy *impairs *Candida albicans *and *Streptococcus mutans* mixed biofilm formation *in vitro*** **”. **We also extend our deep gratitude to the reviewers for providing valuable comments and correction on our paper. We have read and understood the comments from the reviewers. Please find below our point-by-point response to the reviewers' comments and concerns. We hope our additional explanations will be able to answer the reviewers' inquiry.

We strived to improve the quality of our paper based on the guidance and constructive suggestion provided by the reviewers. We hope that the revisions will be sufficient to make our paper worthy for publication in F1000 Research. We would like to thank you for your hearty support and willingness to find the parts of our paper needing corrections or improvement. Thank you again for your consideration, suggestions, and insightful feedback in order to greatly improve our paper.

Sincerely yours,

Authors: Siska Septiana, Christofora Hanny Wijaya*, Boy Muchlis Bachtiar and Nancy Dewi Yuliana.

**Reviewer Comments and Author Responses**

**Section: Introduction**

**Comment 1:** *It would be best if the authors can include the objective/s and the hypothesis of the study in the introduction section.*

**Response to reviewer’s comments 1**:

Thank you very much, we agree with your suggestion. We have emphasized our sentence in the respective paragraph as follows (third sentence in the last paragraph of the introduction section):

*“As C. albicans and S. mutans have been noted for their synergistic relationship* *^5^* *^,^* *^7^* *^,^* *^8^* *^,^* *^10^* *, we evaluated the capacity of CC to impair their symbiotic interaction in this study”* into **“This functional candy may have interfered with their synergistic relationship in dual-species biofilm** **^5^** **^,^** **^7^** **^,^** **^8^** **^,^** **^10^** **. Therefore, this study aimed to evaluate the capacity of CC to impair their symbiotic interaction** **.”...**

**Section: Methodology**

**Comment 2:**

*Microbial strains and MCEO sample: Did C. albicans was grown in aerobic or anaerobic?*

**Response to reviewer’s comments 2**:

Thank you for your comments on this issue. We prepare the separate culture of microbial strain based on the previous reports (Ikono et al. 2019). As mention in the literature, the *C. albicans *as single biofilm was cultured aerobically, after mixed with *S. mutans *they were cultured anaerobically (CO­ _2 _concentration up to 10%) *.* *C. albicans *commonly have optimal growth in aerobic condition, however, it also can grow in anaerobic conditions (elevated CO­ _2 _concentration) (Anand and Prasad 1991; Thein et al. 2007).

**Comment 3:** *For MCEO Pulau Buru, what is the temperature in the boiler? If it is 100 degrees Celcius, won't it affect the active compound of the extract? Is this the same method used for the other extracts? If yes, it would be great if you mention that in the paragraph.*

**Response to reviewer’s comments 3**:

Thank you for the comments and suggestions. MCEO has been widely reported to have antimicrobial activity (Amri et al. 2012; Rini et al. 2012; Wińska et al. 2019). Related to your concern about the MCEO production, hydrodistillation is the most common method for the essential oil extraction, in which the boiler temperature would possible to achieve 100 °C approximately. It is reasonable that this high temperature might affect several bioactive compounds on the leaves and twigs of the plants. However, the extracted essential oil still had antimicrobial activities due to the presence of various terpene compounds as it has been reported in the above-mentioned publications. Based on our recently published article, it was also known that after passing the high-temperature process in candy making, the important terpene compounds in the essential oil were persisting (Wijaya et al. 2020).

Regarding a similar method that has been used for the other extracts, we have been added the information in the article as suggested by the reviewer ( *last sentence in Microbial strains and MCEO samples*). “….. the residual water. **A similar method was also used for the other extracts** **.** The essential oil is stored in a dark bottle.”

**Comment 4:** *CC preparation*

*Won't 150 degrees Celcius affect your active compound? If that is the method, it would be better if you can put a citation on the method. As the expertise in natural product, it seems the temperature is too high for an extract.  Unless if the method is commonly used in the preparation by the local.*

**Response to reviewer’s comments 4**:

Thank you very much for your comment on this CC preparation. We agree with your opinion, the high temperature might affect several compounds in MCEO. However, our study was concern about **the capacity of CC as functional food after passed this high-temperature process (150 °C) during the preparation, whether the CC still active or not**. Moreover, 1,8-cineole, caryophyllene, and a-terpineol as the highest abundance compounds on MCEO and were predicted as the most responsible compound on the MCEO antimicrobial activity commonly had a higher boiling point than the temperature of CC preparation. It had over 176°C for 1,8-cineole ( https://www.chemicalbook.com/ChemicalProductProperty_EN_CB2853653.htm) and over 200°C for caryophyllene, and a-terpineol ( https://www.chemicalbook.com/ProductChemicalPropertiesCB6229317_EN.htm). Moreover, our preliminary study revealed that most of them were still present in the candy after the heating process (Wijaya et al. 2020).

Regarding the CC preparation method, it has been followed the patented procedure as mention in our paper (Wijaya et al. 2016). Based on our result, this functional food showed the expected antimicrobial capacity against dual-species biofilm.

**Comment 5:** *Mixed biofilm formation*

*When you prepare the mixed biofilm, you did mention that you grew C. albicans in SDB and S. mutans in BHI in equal volume. Did you mix both media to prepare biofilm? If not, please make it clear in your text.*

**Response to reviewer’s comments 5**:

Thank you for your interest in this issue. Yes, we did. 50 µL *C. albicans* in SDB and 50 µL of  *S. mutans* in BHI were inoculated together in the same well to promote initial adhesion. As mention in the article ( *fourth sentence of* *mixed* *biofilm formation section) *After the 90 min incubation time, the medium was discarded followed by washing, then, we added Tryptic Soy Broth (TSB)+ 1% sucrose as the medium for the dual-species growth (Ikono et al. 2019). We have also added the reference.

**Comment 6:** *Mixed biofilm formation*

*Why did you coat the well with fetal bovine serum? Better if you can explain why because that is not the standard method used in polymicrobial biofilms study.*

**Response to reviewer’s comments 6**:

Thank you for your comments regarding our selected method (second sentence in the mixed biofilm formation). Yes, we agree with your opinion. In the dual-species biofilm formation especially for *C. albicans *and * S. mutans*, it is common to use saliva coating. In this *in vitro *study, an ethical clearance to use human saliva was not enclosed. Therefore, we used FBS since either saliva or serum can be used to induce phenotype-associated *C. albicans* biofilm formation (Barbosa et al. 2016; Krzyściak et al. 2017; Rodrigues et al. 2020). We have embedded this additional information in the third sentence as follows:” Similar with saliva, FBS coating aims to induce phenotype-associated *C. albicans* biofilm formation (Barbosa et al. 2016; Krzyściak et al. 2017; Rodrigues et al. 2020).

**Comment 7:** *Mixed biofilm formation*

*For you control G0, you mentioned the biofilm was developed without CC. What is the final volume of the well? Did that remain to 200 uL or 140 uL of TSB? If it was 140 uL, how do you compare with other wells which the total volume is 200 uL?*

**Response to reviewer’s comments 7**:

Thanks for your deep evaluation of this mixed biofilm formation. The final volume for control (G0) was similar to other wells (200 uL). To achieve the equal volume, we added PBS (pH 7) on the control to replace the CC formula. We have added this information as the sixth sentence on this mixed biofilm formation paragraph: *”* *For the untreated control, the formula was replaced by 60 µL sterile phosphate-buffered saline (PBS)“*. We expect that this PBS had no inhibition capacity on the culture. Therefore, as expected, all of the formulae (G1-G5) tested showed a significantly different effect compared to the control.

**Comment 8:** *Morphology analysis of dual-species biofilm formation*

*Why did you choose acrylic as the surface to form a biofilm, and how do you relate this with well that have a different surface?*

**Response to reviewer’s comments 8**:

Thank you so much for your question. We conducted this analysis based on the previously published paper which used a similar surface (acrylic disc) (Barbosa et al. 2016). The use of acrylic disc (on 24-well plates) was one of the common methods for the morphology analysis of microbial biofilm to facilitate the biofilm sample to be analyzed by using SEM.  Moreover, by using SEM, we only focused on the altered morphology between the organisms in control and those treated biofilms (within the group), thus we grew the biofilm on the same surface as mention in the literature.

**Section: Results**

**Comment 9:** *Effect of CC on dual-species biofilm development*

*'Susceptibility' is generally referred to as the antimicrobial activity of the extract. In your research, you are emphasizing on biofilm (Figure 2) and not the antimicrobial effect of the extract.*

**Response to reviewer’s comments 9**:

Thank you so much for your concern. We would like to confirm that in our case, we used the term *susceptibility *to describe the condition of the cultures by the presence of the CC formula. In another word, this term was focus on the object exposed by the extract whether it is susceptible or resistant. However, we have rephrased this term in the whole text as you suggested. we used “ *efficacy”* to describe the measurement of antimicrobial activity of the CC formula to impair the dual-species biofilm formation.

**Comment 10:** *Effect of CC on dual-species biofilm development*

*Figure 1. In the legend, the author mentioned that different letters represent significantly different. What does the author comparing too? Significant compared to what? Better to mention.*

**Response to reviewer’s comments 10**:

Thank you for your review. The statistical analysis on CV assay and MTT assay (Figure 1) was conducted using ANOVA followed by Duncan’s test. In this post hoc analysis, all of the formulae **were** **compared to each other** (G0 to G1, G2, G3, G4, G5; G1 to G2, G3, G4, G5; and so on with similar way) **within the group in 0, 3, and 24 hours**. The significantly different results within the group were then indicated by different letters. We have revised the figure and completed the figure legend with the additional information as suggested.

***“Figure 1.*** *… absorbance at 600nm. **The letters on histogram represented the significantly different values compared to each other formula within the groups **in 0, 3, or 24 hours according to Duncan’s test (p <0.05)…”*

**Comment 11:** *Effect of CC on dual-species biofilm development*

*What is a, b and c represent?  E.g. 'a' in Figure 1A doesn't show significant at 3 hours. The figure seems confusing. I would suggest improving the figure to ensure understanding of readers.  Similar to figure 2.*

**Response to reviewer’s comments 11**:

Similar as the response to the reviewer’s comment no. 10, the use of the different letter (a, b, and c or x, y, and z) was only to describe the significantly different values between the formula (G0-G5) within the group in 0, 3, and 24 hours of incubation time, e.g: in 24 hours the statistic analysis result showed by x, y, and z for the significantly different value among the formulae, whereas “xy” was not significantly different with x and y.

Based on Duncan’s test, G1-G5 in 3 hours incubation was showed by ‘a’ and it was significantly different from the negative control which had ‘b’ (Figure 1A). This was our expected result, in which the exposure of CC after 3 hours could inhibit the biofilm formation effectively. However, different superscript symbol/letter between 0, 3, or 24 hours does not correlate each other since the values were analyzed within the group.

Thank you for your suggestion to revised our figure. Unfortunately, it seems ineffective to separate each incubation time for CV and MTT assay similar to Figure 2. It will need six additional figures for this revision. Therefore, we have revised the symbol/letter on the histogram to differentiate each incubation time (the revised Figure 1). We hope that this revision would be sufficient to enhance the readers' understanding.

(the revised Figure 1 was uploaded separately)

**“Figure 1. **… absorbance at 600nm **. The letters on histogram represented the significantly different values compared to each other formula within the groups in 0, 3, or 24 hours according to Duncan’s test (p <0.05)**…”

**Comment 12:** *Effect of CC on the morphology of dual-species biofilm*

*Figure 3A doesn't look corncob to me. Please read this article: The microbial infection of biomaterials: A challenge for clinicians and researchers. A short review. Journal of Applied Biomaterials & Biomechanics 2005; Vol. 3 no. 1: 1-101*

**Response to reviewer’s comments 12**:

Thank you, we appreciate your deep review on our figure and your attached literature. Figure 3A describes how the growth of the microbes in the first 90 minutes period, before the exposure to the CC formula. Figure 3B showed the biofilm growth after 24 h, whereas figure 3C indicated an interaction between *C. albicans *and *S. mutans* after the exposure of CC. The interaction between those two microbes commonly known as corn-cob like structure (Zijnge et al. 2010). Although Figure 3C still not clearly showed this structure due to the limitation of the light microscopy that we used in the analysis, it indicates that this structure might occur since *S. mutans *was attached to * the C. albicans *hyphae as describe in the figure.

**Comment 13:** *Effect of CC on the morphology of dual-species biofilm*

*What do you mean by microcolonies with reference to the figure? Please explain.*

**Response to reviewer’s comments 13**:

Regarding the SEM figure, there is part of the figure which indicates the presence of microcolonies (no. 5 in Figure 4B). This microcolony was referred to a microscopic colony of *S. mutans *cells without considering its interaction with *C. albicans.*

**Comment 14:** *Effect of CC on the morphology of dual-species biofilm*

*How do you determine significant from SEM image? What was the statistical analysis did you use to claim the significant of your SEM? Did you use image J or any software to measure the biofilm based on your SEM?  If yes, better to mention in your methodology. If not, please remove the 'significant'.*

**Response to reviewer’s comments 14**:

Thank you so much for your correction. We didn’t conduct the quantitative analysis on SEM images. We have removed this term through the whole text as suggested.

**Comment 15:** *Effect of CC on the expression of biofilm-related genes*

*The author mentioned 'upregulated dominantly'. What do you mean by dominantly and compare to what? G0? G1?*

**Response to reviewer’s comments 15**:

Thank you for your review of this case. The mRNA expression level was presented as the relative result of its comparison to control (G0) which had value as 1 (one). After this relative quantification, YWP1 has upregulated tens of times higher than other *C. albicans* specific genes (HWP1 and ALS3), this is what we stated as YPW1 *'upregulated dominantly'* which compares to other genes. As it might be confusing the readers, we have revised this sentence into “…expressed in G1, G2, and G3. However, the expression of *YWP1*, the yeast-specific gene, **was had a higher upregulation in almost all of the CC** **groups** **than** **other specific genes (HWP1 and ALS3)** (Figure 5A).” (third sentence of Result section on paragraph *Effect of *CC *on the expression of biofilm-related* *genes)*

**Comment 16:** *What do you mean by 'to maintain the commensal form of C. albicans'?*

**Response to reviewer’s comments 16**:

Thank you for your interest in this part. *C. albicans* is commonly recognized as the most prominent human commensal fungi which could grow by taking nutrient on the human body without interfering the human body homeostasis. This condition occurs when *C. albicans* is in the yeast state. Unfortunately, the phenotype switching into hyphae often caused this commensal fungus to become a pathogen (Finkel and Mitchell 2011). This morphological change represented several virulence genes which might be induced as mention in the third sentence of our introduction section “The hyphal form is relevant for its virulence as it allows penetration and invasion of epithelial cells (Moyes *et al. *2015)”. Therefore, the exposure of CC on the dual-species biofilm was expected to maintain the yeast state of *C. albicans* so that its commensal properties remain. In other words, the commensal form describes the yeast state of *C. albicans*. This yeast form would suppress its virulence and to minimize the interaction with *S. mutans.*

**Section: Discussion**

**Comment 17:** *The author claims that all CC groups reduce biofilm biomass. What is the author comparing too? Based on my observation, there is no significant difference in total biomass for 24 h and 0 h when all formulation are compared to G0. Please check your statistics.*

**Response to reviewer’s comments 17**:

Thank you for your further review of this data. Our statement was based on Duncan’s test as our statistical approach. As describe in the response no.10-11, this test showed that G1-G5 had different superscript letters with G0 on each groups (0, 3 and 24 h) for the total biofilm which indicated significantly different values have occurred (Figure 1A). It also means that the CC formula significantly affected biofilm formation on 0, 3 and 24 h without comparing the group.

**Comment 18:** *Figure 4D. This doesn't look cocci shape to me. More like bacilli/rod. I hope you did verify your sample prior to the experiment. Attached is the SEM of commonly seen Streptococcus mutans under SEM.*

**Response to reviewer’s comments 18**:

Thank you very much for your deep review of our SEM images. We have verified our sample prior to the analysis. The different shapes with your attached literature might be correlated with the different strains that have been used. We used *S. mutans* serotype c strain, the most isolated strain from dental plaque, which possible to have a different shape with *S. mutans *ATCC 25175 in your attached literature (Lim et al. 2017). Our SEM analysis showed that *S. mutans* serotype c had longer shape than ATCC 25175 strain.

Our analysis method was conducted based on literature (Barbosa et al. 2016), which also used *S. mutans *UA159 (a serotype c strain). In their report, *S. mutans* also had a long shape (showed in Figure 3) similar to our result. Our image also supported by Feldman *et al.* (Feldman et al. 2016) which reported that biofilm was dominated by the long shape of *S. mutans *UA159 (a serotype c strain) which was described in Figure 3 in the literature. Sztajer *et al.* (Sztajer et al. 2014) had a clear visualization of dual-species biofilm of *C. albicans *and *S. mutans *UA159 (Figure 1e-f), in which the long shape *S. mutans* attached to the hyphae of  *C. albicans*. Therefore, it was reasonable that our *S. mutans* strain also have a long shape.

**Comment 19:** *The author mentioned that lower gtfB indicate the fewer binding site. Glucosyltransferases (Gtfs) are enzymes and not a 'binding site'. This is too speculative. Please consider to rephrase or to remove the statement.*

**Response to reviewer’s comments 19**:

Thank you for your correction. We understand your opinion about this issue. Based on our result, the exposure of G2 caused the fewer *gtfB* expression level. This fewer *gtfB* contributed to the fewer extracellular polysaccharide matrix formation, in which this matrix is needed as a mediator of interaction between *C. albicans *and *S. mutans.* Therefore, we have revised our sentence as suggested (fifth sentence in fourth paragraph of Discussion section) into : “…Lower *gtfB *expression indicated **a fewer matrix formation of *S. mutans* which important to form a polymicrobial biofilm **with *C. albicans,* as shown by the CV and MTT assays in this study.”

**Comment 20:** *The author also emphasised a lot on quorum sensing molecule which is not studied in the manuscript. The author also mentioned that QSM is not measured quantitatively. This statement can be misinterpreted none of the section showed the authors have conducted study on QSM either quantitatively or qualitatively. Please consider rephrasing.*

**Response to reviewer’s comments 20**:

Thank you for your response. The quorum-sensing molecule information was added in the text as the most possible mechanism that supports our result based on the literature. Several QSM has been reported to be involved in the dual-species interaction between *C. albicans *and *S. mutans*. We assumed that we should embed this explanation as additional information to enrich the readers' insight related to the dual-species biofilm formation. Although QSM was not measured in our study, these molecules might have a role in their synergism. Therefore, we have revised our last sentence in this QSM paragraph into “…QSMs in the mixed biofilm was not measured quantitatively or qualitatively in our study. However, this assumption needs to be studied further.”

References

- Amri I, Mancini E, de Martino L, Marandino A, Lamia H, Mohsen H, Bassem J, Scognamiglio M, Reverchon E, de Feo V. 2012. Chemical composition and biological activities of the essential oils from three Melaleuca species grown in Tunisia. *Int. J. Mol. Sci.* 13(12):16580–16591. doi:10.3390/ijms131216580.
- Anand S, Prasad R. 1991. Growth and Respiration Characteristics of Candida albicans. In Candida Albicans, R. Prasad (Ed. by), Springer, Berlin, Heidelberg, pp. 46–61.
- Barbosa JO, Rossoni RD, Vilela SFG, De Alvarenga JA, Velloso MDS, De Azevedo Prata MC, Jorge AOC, Junqueira JC. 2016. Streptococcus mutans can modulate biofilm formation and attenuate the virulence of Candida Albicans. *PLoS One* 11(3):1–16. doi:10.1371/journal.pone.0150457.
- Feldman M, Ginsburg I, Al-quntar A, Steinberg D. 2016. Thiazolidinedione-8 Alters Symbiotic Relationship in C . albicans - S . mutans Dual Species Biofilm. *Front. Microbiol.* 7(140):1–12. doi:10.3389/fmicb.2016.00140.
- Finkel JS, Mitchell AP. 2011. Genetic control of Candida albicans biofilm development. *Nat. Rev. Microbiol.* 9(2):109–118. doi:10.1109/isdrs.2005.1596138.
- Ikono R, Vibriani A, Wibowo I, Saputro KE, Muliawan W, Bachtiar BM, Mardliyati E, Bachtiar EW, Rochman NT, Kagami H, et al. 2019. Nanochitosan antimicrobial activity against Streptococcus mutans and Candida albicans dual ‑ species biofilms. *BMC Res. Notes* 12(383):1–7. doi:10.1186/s13104-019-4422-x.
- Krzyściak W, Kościelniak D, Papież M, Vyhouskaya P, Zagórska-Świeży K, Kołodziej I, Bystrowska B, Jurczak A. 2017. Effect of a Lactobacillus salivarius probiotic on a double-species streptococcus mutans and Candida albicans caries biofilm. *Nutrients* 9(11):1–23. doi:10.3390/nu9111242.
- Lim JH, Song SH, Park HS, Lee JR, Lee SM. 2017. Spontaneous detachment of Streptococcus mutans biofilm by synergistic effect between zwitterion and sugar alcohol. *Sci. Rep.* 7(1):1–9. doi:10.1038/s41598-017-08558-x.
- Rini P, Ohtani Y, Ichiura H. 2012. Antioxidant, anti-hyaluronidase and antifungal activities of Melaleuca leucadendron Linn. leaf oils. *J. Wood Sci.* 58(5):429–436. doi:10.1007/s10086-012-1270-x.
- Rodrigues ME, Gomes F, Rodrigues CF. 2020. Candida spp./Bacteria Mixed Biofilms. *J. Fungi* 6(5):1–29.
- Sztajer H, Szafranski SP, Tomasch J, Reck M, Nimtz M, Rohde M, Wagner-Dobler I. 2014. Cross-feeding and interkingdom communication in dual-species biofilms of Streptococcus mutans and Candida albicans. *Int. Soc. Microb. Ecol.* 81–16. doi:10.1038/ismej.2014.73.
- Thein ZM, Samaranayake YH, Samaranayake LP. 2007. In vitro biofilm formation of Candida albicans and non-albicans Candida species under dynamic and anaerobic conditions. *Arch. Oral Biol.* 52(8):761–767. doi:10.1016/j.archoralbio.2007.01.009.
- Wijaya CH, Sari BR, Bachtiar BM. 2020. The potency of cajuputs candy in maintaining the competitive capacity of Streptococcus sanguinis upon Streptococcus mutans. *J. Funct. Food Nutraceutical* 1(2):55–65. doi:10.33555/jffn.v1i2.29.
- Wijaya CH, Fickie A, Nurramdhan IF, Bachtiar BM, Afandi FA, Indariani S. 2016. The Composition of Cajuputs Candy which inhibits the growth of dental caries microbes. Indonesia; IDP000040695.
- Wińska K, Mączka W, Łyczko J, Grabarczyk M, Czubaszek A, Szumny A. 2019. Essential Oils as Antimicrobial Agents—Myth or Real Alternative? *Molecules* 24(11):1–20. doi:10.3390/molecules24112130.
- Zijnge V, Leeuwen MBM Van, Degener JE, Abbas F, Thurnheer T, Gmu R. 2010. Oral Biofilm Architecture on Natural Teeth Oral Biofilm Architecture on Natural Teeth. *PLoS One* 5(2):1–9. doi:10.1371/journal.pone.0009321.
